# National Survey of Real‐World Australian Treatment Patterns for Patients With Very‐Early‐To Intermediate‐Stage Hepatocellular Carcinoma

**DOI:** 10.1002/cam4.70722

**Published:** 2025-02-28

**Authors:** Stuart K. Roberts, Ammar Majeed, Kiran Rasaratnam, John K. Olynyk, Nicholas Shackel, Marnie Wood, Simone I. Strasser, Alan Wigg

**Affiliations:** ^1^ Department of Gastroenterology, Alfred Health Alfred Health Melbourne Victoria Australia; ^2^ Department of Medicine, Central Clinical School Monash University Melbourne Australia; ^3^ Associate Dean, Curtin Medical School Curtin University Bentley Western Australia Australia; ^4^ Department of Gastroenterology Launceston General Hospital Launceston Tasmania Australia; ^5^ Department of Gastroenterology Royal Brisbane and Womens Hospital Brisbane Queensland Australia; ^6^ AW Morrow Gastroenterology and Liver Centre Royal Prince Alfred Hospital Sydney NSW Australia; ^7^ Hepatology and Liver Transplant Medicine Unit Southern Adelaide Local Health Network Bedford Park South Australia Australia; ^8^ College of Medicine and Public Health Southern Adelaide Local Health Network Bedford Park South Australia Australia

**Keywords:** early stage, hepatocellular carcinoma, intermediate stage, survey, treatment

## Abstract

**Background/Purpose of the Study:**

The treatment landscape for very early to intermediate stage hepatocellular carcinoma (HCC) is rapidly evolving, with new data and treatments emerging in recent years. There is a lack of data on current patterns of management for very early to intermediate stage HCC in Australian clinical practice and the role of newly emerging treatment options.

**Methods:**

Multidisciplinary specialists involved in HCC management (*N* = 86) participated in one of six state‐based meetings across Australia. Specialists were surveyed on their preferred management approaches at key clinical decision points for four patient case studies ranging from very early to intermediate stage HCC.

**Results:**

Preferred management strategies for each of the patient case studies were largely consistent with current Australian HCC recommendations in relation to surveillance, diagnosis, and treatment of HCC although the preferred initial treatment selection varied considerably within and between hepatologists and other craft groups. There was, however, growing interest in emerging treatments, including stereotactic ablative body radiotherapy (SABR) for early stage HCC and systemic treatments used as adjuvant therapy or in combination with locoregional therapy in early and intermediate‐stage HCC. However, many participants required more data on these treatment modalities before incorporating them into routine clinical practice.

**Conclusion:**

The heterogeneity of (very) early to intermediate‐stage HCC patients and the increasing number of available treatment options means clinical decision‐making, including treatment selection, is becoming more complex and diverse. More data are required to define the role of SABR and systemic therapies in very early to intermediate stage HCC before being adopted as standard of care in Australia.

## 1 Introduction

Primary liver cancer is one of the most commonly diagnosed cancers in Australia and the sixth leading cause of cancer‐related mortality [1]. Hepatocellular carcinoma (HCC) is the most common type of primary liver cancer, comprising 75% of Australian cases [1]. The incidence of HCC continues to rise, with HCC mortality rates increasing faster than any other cancer type between 2001 and 2021 [1, 2].

Management of HCC is complex and requires a multidisciplinary approach due to the many factors that must be considered, including the presence of cirrhosis; liver function; the size, location, and burden of HCC; the patient's age, comorbidities, and fitness; multiple treatment modalities; and patient preferences. The Barcelona Clinic Liver Cancer (BCLC) staging system takes many of these factors into account and is endorsed by most international HCC guidelines, including Australian HCC consensus recommendations [3, 4, 5, 6].

Very early stage to intermediate stage HCC (BCLC‐0 to BCLC‐B) covers the spectrum of non‐metastatic HCC [4]. This represents a highly heterogeneous group of patients in terms of tumour burden, liver function, clinical characteristics, and recommended treatment options [4]. Current recommendations for initial treatment for this patient group include curative options such as hepatic resection, liver transplant, and ablation; and non‐curative locoregional therapies (LRT) such as transarterial chemoembolisation (TACE) and selective internal radiation therapy (SIRT). Systemic therapy is generally reserved for when LRT is contraindicated or likely to be ineffective [3, 4, 5, 6].

The treatment landscape for HCC is rapidly evolving, with new data and new treatment approaches such as stereotactic ablative body radiotherapy (SABR) emerging and a growing interest in the role of systemic therapy at earlier stages of HCC in combination with LRT [5, 6, 7, 8, 9]. There is currently a lack of data on patterns of management for very early to intermediate stage HCC in clinical practice across Australia and the impact of newly emerging treatment options on treatment approaches. This study aimed to describe current Australian real‐world clinical management patterns for surveillance, diagnosis, and treatment of patients with very early to intermediate‐stage HCC.

## 2 Methodology

A series of six state‐based meetings was held with specialist healthcare professionals across a range of disciplines involved in the management of HCC. Specialists were selected based on recognised expertise in one of the various disciplines relating to HCC management, including gastroenterology/hepatology, hepatobiliary surgery, interventional/diagnostic radiology, radiation/medical oncology, nursing, and pathology. All states and territories of Australia were represented except for the Northern Territory. Specialists were selected from both metropolitan and regional centres to ensure representative treatment practices were captured.

Each meeting focused on the same four de‐identified patient case studies ranging from very‐early‐ to intermediate‐stage HCC, which included detailed patient demographics, clinical history, laboratory findings, imaging reports, treatment approaches, and outcomes (Table 1). At pre‐defined clinical decision points for each clinical scenario, specialists were surveyed on their preferred management approaches via an online survey platform. Survey questions included a mix of multiple‐choice questions where specialists were required to select their preferred management option and open‐ended questions that asked specialists to provide further information or a rationale for their treatment approach. Management options provided in the multiple‐choice questions were based on Australian consensus recommendations for the management of HCC (Table S1) [4]. Facilitated group discussions were conducted following survey questions to provide more context on the preferred treatment approaches. All discussions were audio recorded and transcribed verbatim with participant consent.

**TABLE 1:** Very early to intermediate stage HCC patient case studies.

**Case study 1: Very‐early‐stage HCC (BCLC‐0)**
Clinical history: 74‐year‐old female with multiple comorbidities including cardiomyopathy, UTI, falls, cognitive decline, bronchiectasis and history of AUD (abstinent 5 months). Currently living in an aged care facility. Presentation: 11 mm S3 lesion HCC (LiRADS5) found on surveillance ultrasound; features of cirrhosis; splenomegaly; no definite intra‐abdominal varices. Laboratory findings: AFP 3 IU/mL; ALP 174 IU/L; GGT 346 IU/L; bilirubin 11 μmol/L; albumin 34 g/L; Hb 88 g/dL; WBC 4.8; Plt 178×10^9^/L; INR 1.1.
**Case study 2: Early stage HCC (BCLC‐A)**
Clinical history: 83‐year‐old male with multiple comorbidities, including compensated Child‐Pugh A cirrhosis (ETOH/MAFLD), type 2 diabetes, hypertension, ischaemic heart disease, peripheral vascular disease, abdominal aortic aneurysm, CKD Stage 3A, and AUD (60 g/d). Presentation: Incidental 53 mm S4A HCC (LiRADS5) identified during AAA surveillance. Laboratory findings: AFP 5 IU/mL; Ca19‐9 40 U/mL; bilirubin μmol/L; albumin 39 g/L; ALP 315 IU/L; GGT 654 IU/L; ALT 49 IU/L; Plt 110 ×10^9^/L; INR 1.1.
**Case study 3: Intermediate‐stage HCC (BCLC‐B)**
Clinical history: 57‐year‐old male crane operator with a history of HCV (SVR in 2015) and HBV, presumed cirrhosis following pre‐DAA elastography, hypertension and moderate alcohol use. Presentation: 8 cm S7 lesion (LiRADS 5) with small satellite lesions; no macrovascular invasion; no metastases. Laboratory findings: AFP 9113 IU/mL.
**Case study 4: Intermediate‐stage multifocal bilobar HCC (BCLC‐B)**
Clinical history: 68‐year‐old female with a history of HCV (SVR in 2016) and multiple comorbidities, including compensated cirrhosis with clinically significant portal hypertension, peripheral neuropathy, frequent falls, congenital solitary kidney and AUD (60 g/d). History of several years of recurrent HCC lesions treated with MWA and/or cTACE with complete response. Lost to follow‐up after 18‐month hospitalisation following a fall with fracture and eventual below‐knee amputation. Presentation: Multi‐focal, bilobar HCC with 5 lesions identified in S2, S5 and S7. Laboratory findings: AFP 1900 IU/mL; Child‐Pugh A; ALBI Grade 1; MELD 8; Plt 150×10^9^/L.

Abbreviations: AAA, abdominal aortic aneurysm; AFP, alpha fetoprotein; ALBI: albumin‐bilirubin; ALP, alkaline phosphatase; ALT, alanine aminotransferase; AUD, alcohol use disorder; BCLC, Barcelona Clinic Liver Cancer; Ca19‐9, carbohydrate antigen 19–9; CKD, chronic kidney disease; cTACE, conventional transarterial chemoembolisation; DAA, direct‐acting antiviral; ETOH, ethanol; GGT, gamma‐glutamyl transferase; Hb, haemoglobin; HBV/HCV, hepatitis B/C virus; HCC, hepatocellular carcinoma; INR, international normalised ratio; LiRADS, Liver Imaging Reporting and Data System; MAFLD, metabolic associated fatty liver disease; MELD, model for end‐stage liver disease; MWA, microwave ablation; Plt, platelets; SVR, sustained virologic response; UTI, urinary tract infection; WBC, white blood cells.

Quantitative and qualitative data were collected from the surveys and analyzed for key aspects of very‐early‐to‐intermediate‐stage HCC management, including surveillance, diagnosis, initial management, and management of residual and progressive disease. Descriptive statistics (frequencies and percentages) were used to analyze survey responses in each category. For open‐ended survey responses, themes were identified and grouped before analysis. Responses from gastroenterologists/hepatologists were analyzed separately from responses from other specialists due to their involvement in every aspect of patient care from surveillance/diagnosis through to best supportive care. The current study is exempt from ethical approval, so no Ethics Committee approval was obtained from The Alfred Hospital Ethics Committee.

## 3 Results

A total of 79 specialists participated in meetings across six Australian states. Specialists had expertise across a broad range of specialties related to HCC management, with the makeup of the overall expert panel generally reflecting that of a typical HCC multidisciplinary team (MDT) (Table 2). Most specialists (78%) were based at metropolitan public hospitals, with 10% practicing at regional hospitals, either solely or in addition to their practice at metropolitan hospitals. Most specialists (74%) had been managing patients with HCC for at least 6 years.

**TABLE 2:** Survey participant characteristics.

**Primary speciality**	
Total respondents	*N* = 79
Gastroenterologist/hepatologist	34 (43%)
Hepatobiliary surgeon	8 (10.1%)
Interventional radiologist	9 (11.4%)
Diagnostic radiologist	0 (0%)
Medical oncologist	7 (8.9%)
Radiation oncologist	10 (12.7%)
HCC nurse	10 (12.7%)
Pathologist	1 (1.3%)
**Medical facility**	
Total respondents*	*N* = 73
Metropolitan public hospital	64 (78%)
Metropolitan private hospital	10 (12%)
Regional hospital	8 (10%)
Rural hospital	0 (0%)
Other	0 (0%)
**Years managing HCC**	
Total respondents	*N* = 77
1–5 years	20 (26%)
6–10 years	26 (34%)
11–15 years	14 (18%)
16–20 years	9 (12%)
> 20 years	8 (10%)

*Note:* Percentages may not add to 100% due to rounding.

Abbreviation: HCC, hepatocellular carcinoma.

* Some respondents practiced at more than one medical facility.

### 3.1 HCC Surveillance

All gastroenterologists/hepatologists, when questioned regarding Case 1, routinely perform HCC surveillance for at‐risk patients using ultrasound (US) every 6 months with or without serum alpha fetoprotein (AFP). A smaller proportion of other specialists (53%) perform surveillance using the same methods; the remainder are not involved in managing patients pre‐HCC diagnosis, and so HCC surveillance is not within their scope of practice (Table 3).

**TABLE 3:** Patterns of HCC surveillance for at‐risk patients.

	Gastroenterologist/hepatologist	Other	Total
Method and frequency of surveillance, *n* (%)			
Total respondents	*N* = 34	*N* = 49	*N* = 83
US + AFP every 6 months	**30 (88%)**	**18 (37%)**	**48 (58%)**
US every 6 months	4 (12%)	8 (16%)	12 (15%)
US every 12 months	0 (0%)	0 (0%)	0 (0%)
US + AFP every 12 moths	0 (0%)	0 (0%)	0 (0%)
CT scan +/− AFP	0 (0%)	0 (0%)	0 (0%)
This is outside my scope of practice	0 (0%)	23 (47%)	23 (28%)
Implementation of age cut‐off for surveillance, *n* (%)			
Total respondents	*N* = 34	*N* = 43	*N* = 77
Depends on patient comorbidities	**30 (88%)**	**25 (58%)**	**55 (71%)**
No	0 (0%)	10 (23%)	10 (13%)
Yes	1 (3%)	1 (2%)	2 (3%)
Not applicable	3 (9%)	7 (16%)	10 (13%)
Specific age cut‐off for surveillance, *n* (%)			
Total respondents	*N* = 23	*N* = 22	*N* = 45
No age cut‐off	**16 (70%)**	**15 (68%)**	**31 (69%)**
> 90 years	2 (9%)	4 (18%)	6 (13%)
> 80 years	4 (17%)	2 (9%)	6 (13%)
> 70 years	1 (4%)	1 (5%)	2 (4%)

*Note:* The most frequently selected responses are shown in bold. Percentages may not add to 100% due to rounding.

Abbreviations: AFP, alpha fetoprotein; CT, computed tomography; US, ultrasound.

Approximately 70% of specialists do not use a single age cut‐off for surveillance; additional factors are also considered, such as comorbidities and overall fitness for treatment that may influence whether early HCC detection is likely to prolong a patient's life expectancy. Specialists noted that their clinics are currently not able to cope with the volume of patients requiring HCC surveillance. Most agreed there is a role for primary care clinicians in performing surveillance for at‐risk patients. However, the reliability of primary care surveillance was a concern due to high GP turnover, lack of GP education, and challenges in ensuring patients have HCC screening performed. Several clinics have successfully implemented nurse‐led HCC surveillance, but funding remains a challenge.

### 3.2 HCC Diagnosis

Almost all specialists (99%) do not routinely biopsy lesions < 2 cm with radiological features characteristic of HCC to confirm diagnosis, as lesion histology is unlikely to change their treatment approach. However, if the lesion is treated with microwave ablation (MWA), then biopsy may be performed at the time of treatment. In total, 29% of specialists never perform biopsies for these lesions (Table 4).

**TABLE 4:** Diagnosis of HCC.

	Gastroenterologist/hepatologist	Other	Total
Biopsy to confirm diagnosis of < 2 cm lesion with radiological features of HCC, *n* (%)			
Total respondents	*N* = 34	*N* = 42	*N* = 76
Always	0 (0%)	1 (2%)	1 (1%)
Sometimes	4 (12%)	5 (12%)	9 (12%)
Occasionally	3 (9%)	7 (17%)	10 (13%)
Rarely	**14 (41%)**	**20 (48%)**	**34 (45%)**
Not at all	13 (38%)	9 (21%)	22 (29%)
Point at which biopsy is performed, *n* (%)			
Total respondents	*N* = 27	*N* = 39	*N* = 66
Pre‐treatment	0 (%)	8 (21%)	13 (20%)
At the time of treatment	**27 (100%)**	**31 (80%)**	**53 (80%)**
Management of very small LiRADS 4/5 lesions with threshold growth but no washout, *n* (%)			
Total respondents	*N* = 25	*N* = 40	*N* = 65
Repeat imaging in 3 months	**21 (84%)**	**34 (85%)**	**55 (85%)**
Ablation	4 (16%)	5 (13%)	9 (14%)
Other	0 (0%)	1 (3%)	1 (2%)
Repeat imaging in 6 months	0 (0%)	0 (0%)	0 (0%)
SABR	0 (0%)	0 (0%)	0 (0%)
Resection	0 (0%)	0 (0%)	0 (0%)

*Note:* The most frequently selected responses are shown in bold. Percentages may not add to 100% due to rounding.

Abbreviations: HCC, hepatocellular carcinoma; LiRADS, Liver Imaging Reporting and Data System; SABR, stereotactic ablative body radiotherapy.

Over half of specialists (56%) did not think liver biopsy will play an increasing role in diagnosing very‐early‐stage HCC over the next 5–10 years. Despite more targeted systemic therapies potentially becoming available, specialists did not consider them likely to be used at this stage of the disease as definitive treatments are already available. The remaining specialists thought biopsy could become more important for risk stratification, molecular targeting of treatments, or for research purposes.

For very small lesions with radiologic features generally consistent with HCC (LiRADS 4) and threshold growth but no washout, 85% of specialists would wait and repeat imaging in 3 months (Table 4). Most of the remaining specialists (14%) would perform MWA without confirming the diagnosis, except for one specialist who would treat with SABR and one specialist who responded “Other”. Neither of these specialists provided further details on their choice of treatment.

### 3.3 HCC Treatment

#### 3.3.1 Very Early Stage HCC

For very early stage HCC (BCLC‐0) in a patient with multiple comorbidities and advanced age, more than half of specialists (52%) would initially treat the lesion with MWA with curative intent (Table 5) [4]. These specialists considered MWA preferable over hepatic resection due to the patient's comorbidities and age. In contrast, 25% of specialists would not treat immediately, instead repeating imaging in 3 months. Other approaches included SABR to avoid general anaesthetic, or no intervention if the patient's life expectancy was low. Of the 17% of specialists who selected “Other” most would seek more information before making a treatment decision, for example, assessing the patient's comorbidities, fitness, and treatment preferences.

**TABLE 5:** Management of patients in very early to intermediate stage HCC.

Treatment modality, *n* (%)	Very‐early‐stage HCC (BCLC‐0)			Large early‐stage HCC (BCLC‐A)			Intermediate‐stage HCC (BLCL‐B)		
	Gastroent‐erologist/hepatologist	Other	Total	Gastroent‐erologist/hepatologist	Other	Total	Gastroent‐erologist/hepatologist	Other	Total
Initial treatment									
Total respondents, *N*	29	50	79	32	42	74	30	41	71
Repeat imaging in 3 months	5 (17%)	15 (30%)	20 (25%)	0 (0%)	0 (0%)	0 (0%)	0 (0%)	0 (0%)	0 (0%)
Liver biopsy	0 (0%)	2 (4%)	2 (3%)	0 (0%)	0 (0%)	0 (0%)	0 (0%)	0 (0%)	0 (0%)
Hepatic resection	0 (0%)	0 (0%)	0 (0%)	1 (3%)	2 (5%)	3 (4%)	7 (23%)	8 (20%)	15 (21%)
MWA	**20 (69%)**	**21 (42%)**	**41 (52%)**	0 (0%)	0 (0%)	0 (0%)	0 (0%)	0 (0%)	0 (0%)
TACE/SIRT	0 (0%)	0 (0%)	0 (0%)	**15 (47%)**	17 (41%)	**32 (43%)**	**17 (57%)**	**22 (54%)**	**39 (55%)**
SABR	1 (3%)	2 (4%)	3 (4%)	10 (31%)	**18 (43%)**	28 (38%)	2 (7%)	0 (0%)	2 (3%)
TACE/SIRT + ablation	0 (0%)	0 (0%)	0 (0%)	2 (6%)	3 (7%)	5 (7%)	0 (0%)	6 (15%)	6 (9%)
Liver transplantation	0 (0%)	0 (0%)	0 (0%)	0 (0%)	0 (0%)	0 (0%)	0 (0%)	1 (2.4%)	1 (1.4%)
Other	3 (10%)	10 (20%)	13^a^ (17%)	4 (13%)	2 (5%)	6^b^ (8%)	4 (13%)	4 (10%)	8^c^ (11%)
Use of systemic therapy as initial treatment for intermediate‐stage HCC with large tumour burden									
Total respondents, *N*				—	—	—	25	41	66
Yes				—	—	—	**15 (60%)**	**18 (44%)**	**33 (50%)**
No				—	—	—	10 (40%)	10 (24%)	20 (30%)
Outside area of expertise				—	—	—	0 (0%)	13 (32%)	13 (20%)
Role for adjuvant therapy after complete response in high‐risk patients									
Total respondents, *N*				28	42	70	27	41	68
Yes				7 (25%)	15 (36%)	22 (31%)	**12 (44%)**	9 (22%)	21 (31%)
No				**21 (75%)**	**27 (64%)**	**48 (69%)**	**12 (44%)**	**17 (42%)**	**29 (43%)**
Outside area of expertise^d^				N/A	N/A	N/A	3 (11%)	15 (37%)	18 (27%)
Management of residual disease in early‐stage HCC									
Total respondents, *N*				32	41	73	—	—	—
Continue to monitor				**17 (53%)**	**24 (59%)**	**41 (57%)**	—	—	—
TACE/SIRT				3 (9%)	8 (20%)	11 (15%)	—	—	—
Depends on patient preferences				3 (9%)	6 (15%)	9 (13%)	—	—	—
Further SABR				3 (9%)	2 (5%)	5 (7%)	—	—	—
Other				5 (16%)	0 (0%)	5 (7%)	—	—	—
TACE/SIRT + ablation				1 (3%)	1 (2%)	2 (3%)	—	—	—
MWA				0 (0%)	0 (0%)	0 (0%)	—	—	—
Management of progressive disease post TACE									
Total respondents, *N*				—	—	—	30	39	69
Systemic therapy				—	—	—	**20 (67%)**	**19 (49%)**	**39 (57%)**
SIRT				—	—	—	7 (23%)	9 (23%)	16 (23%)
Repeat DEB‐TACE to both lesions				—	—	—	2 (7%)	8 (21%)	10 (15%)
Repeat DEB‐TACE to target lesion first				—	—	—	1 (3%)	1 (3%)	2 (3%)
SABR				—	—	—	0 (0%)	2 (5%)	2 (3%)
Best supportive care				—	—	—	0 (0%)	0 (0%)	0 (0%)
Other				—	—	—	0 (0%)	0 (0%)	0 (0%)
Management of significant residual disease after multiple rounds of TACE									
Total respondents, *N*				—	—	—	27	38	65
Systemic therapy				—	—	—	**22 (82%)**	**29 (76%)**	**51 (79%)**
SIRT				—	—	—	4 (15%)	9 (24%)	13 (20%)
Ablation				—	—	—	1 (4%)	0 (0%)	1 (2%)
Resection				—	—	—	0 (0%)	0 (0%)	0 (0%)
Management after complete response post TACE									
Total respondents, *N*				—	—	—	26	33	59
Monitor with 3‐monthly multiphase CT/MRI				—	—	—	**20 (77%)**	**31 (94%)**	**51 (86%)**
Other				—	—	—	4 (15%)	1 (3%)	5 (9%)
Systemic therapy				—	—	—	1 (4%)	1 (3%)	2 (3%)
SABR to large lesion				—	—	—	1 (4%)	0 (0%)	1 (2%)
Liver resection				—	—	—	0 (0%)	0 (0%)	0 (0%)
SIRT				—	—	—	0 (0%)	0 (0%)	0 (0%)
Repeat TACE				—	—	—	0 (0%)	0 (0%)	0 (0%)

Abbreviations: BCLC, Barcelona Clinic Liver Cancer; CT, computed tomography; DEB‐TACE, drug‐eluting bead transarterial chemoembolisation; HCC, hepatocellular carcinoma; MRI, magnetic resonance imaging; MWA, microwave ablation; SABR, stereotactic ablative body radiotherapy; SIRT, selective internal radiation therapy; TACE, transarterial chemoembolization.

*Note:* The most frequently selected responses are shown in bold.

^a^ Other = observation due to co‐morbidities (*n* = 12), clinical trial (*n* = 1).

^b^ Other = TACE + SABR (*n* = 2), MDT evaluation first (*n* = 3), systemic treatment (*n* = 1).

^c^ Other = Systemic treatment (*n* = 3), combination systemic + TACE or SABR (*n* = 4), TACE + SABR (*n* = 1).

^d^ This was not provided as an option in the multiple‐choice question for the BCLC‐A patient case study.

If a patient presenting with a similar disease was otherwise fit and well, most specialists (> 90%) would consider treating the lesion with MWA or hepatic resection, depending on individual patient factors and preferences. The remainder of specialists would wait and repeat imaging in 3 months due to the lesion's small size and low risk of progression.

#### 3.3.2 Early Stage HCC

##### 3.3.2.1 Initial Management

For early‐stage HCC (BCLC‐A) in a patient unsuitable for resection or MWA due to size and location, the preferred initial treatment options were TACE/SIRT (43%) or SABR (38%) (Table 5). Specialists who selected TACE noted that it is the standard of care for the presented patient profile according to Australian consensus recommendations [4]. Reasons for selecting SIRT as an alternative were that it is single‐session and considered to be better tolerated with similar efficacy. As TACE/SIRT is generally not curative, a small proportion of specialists (7%) would perform TACE/SIRT plus ablation. Specialists who selected SABR considered it to have curative potential, with lower toxicity compared with TACE.

Specialists would generally not change their treatment approach if the lesion was located more peripherally; however, for a younger patient (aged < 60 years), most specialists agreed that they would consider a referral for liver transplantation.

##### 3.3.2.2 Residual Disease

For a residual lesion (LiRADS‐5) detected 6 months post‐SABR (the treatment given in case 2), over half (57%) of specialists would continue to monitor the lesion. Radiologists noted that size‐based imaging criteria such as the modified Response Evaluation Criteria in Solid Tumours (mRECIST) are not appropriate for monitoring response to SABR during the first 6–12 months after treatment as lesions may continue to shrink over this time [10]. Other specialists (13%) felt that further treatment would depend on individual patient preferences (Table 5). Overall, 7% of specialists would perform further SABR. Notably, 12% of specialists regularly use SABR in this context in their clinical practice.

In total, 69% of specialists considered that adjuvant systemic therapy does not currently have a role after a complete response in early‐stage HCC, primarily due to insufficient data as well as a lack of reimbursed options in Australia. Those who did see a role for adjuvant systemic therapy noted results from the IMbrave‐050 study, which showed that adjuvant atezolizumab‐bevacizumab following curative MWA or hepatic resection was associated with significantly improved recurrence‐free survival (RFS) compared with active surveillance [7].

#### 3.3.3 Intermediate‐Stage HCC

##### 3.3.3.1 Initial Management

For patients with intermediate‐stage HCC (BCLC‐B) who are not eligible for curative treatments including transplant, 55% of specialists would treat with TACE or SIRT, with the aim of downstaging to meet liver transplantation criteria (Table 5). These specialists considered hepatic resection inappropriate for patients at high risk for metastasis (e.g., high AFP) and recurrence. In contrast, 21% of specialists selected hepatic resection, noting it is the only curative option for this patient profile. Specialists who selected “Other” would use systemic therapy such as atezolizumab‐bevacizumab, also with the intent of downstaging prior to transplant or LRT.

For patients with high tumour burden, 50% of specialists currently consider systemic therapy as the initial treatment if LRT is either contraindicated or deemed likely to be ineffective. They also noted there is a limited window of opportunity to use systemic therapy as patients must have Child‐Pugh‐A (CP‐A) liver function to meet Australian HCC guideline criteria for systemic therapy and Pharmaceutical Benefits Scheme (PBS) reimbursement criteria [4]. The remaining specialists have not used systemic therapy in this setting due to the lack of available data.

##### 3.3.3.2 Progressive Disease

For patients with progressive disease following initial TACE who have multiple comorbidities and preserved liver function, 57% of specialists would repeat TACE/SIRT if the tumor burden was not too high and the original lesion had responded to TACE/SIRT (Table 5). For multifocal, bilobar disease, 57% of specialists would use systemic therapy, noting the efficacy of TACE is low in this context. However, 30% of specialists would use TACE/SIRT if the lesion had previously responded. For more advanced liver disease (e.g., CP‐B8), most specialists (78%) would recommend best supportive care, while the remainder would use systemic therapy (Table S2). Specialists noted that the possibility of liver function loss from multiple rounds of TACE must be considered, as this may preclude a patient from receiving future systemic therapy.

Adjuvant systemic therapy after a complete response to LRT in BCLC‐B HCC was favored by 31% of specialists, while 43% did not believe there is currently a role for adjuvant systemic therapy in this context (Table 5). All specialists agreed that more data are required for a more definitive understanding of the role of adjuvant systemic therapy in intermediate‐stage HCC.

## 4 Discussion

In this real‐world Australian survey of management patterns for very early to intermediate stage HCC, specialists' approaches to surveillance, diagnosis, and management were largely consistent with Australian and international recommendations [3, 4, 5, 6]. However, opinions were diverse in relation to preferred initial treatment selection among hepatologists and other craft groups, and there was growing interest in emerging treatment approaches including SABR and SIRT as alternative LRTs, as well as adjuvant systemic therapy following complete response to LRT, and the use of first‐line systemic therapy for select patients with intermediate‐stage HCC [10, 11, 12].

For initial management of early‐stage HCC where hepatic resection, ablation, or transplant are not appropriate, preferred treatment options were TACE/SIRT or SABR. The Australian HCC consensus statement recommends TACE in this patient group, and while SABR is not recommended for routine use due to limited data, it is recognized as an emerging treatment option that may be considered for select patients [4]. Nonetheless, the use of SABR for HCC is increasing in Australia and globally [10, 13, 14, 15]. In this study, despite 38% of specialists selecting SABR as their preferred treatment approach, only 12% reported that SABR is usually used at their treatment centres for early‐stage HCC where other curative options are contraindicated. It is possible that the availability of SABR expertise at different HCC treatment centres, or lack thereof, may have influenced some specialists' treatment preferences.

Inadequate data on the efficacy and safety of SABR in HCC was identified as a key unmet need by specialists. The body of evidence supporting the use of SABR in early‐to‐intermediate‐stage HCC is growing, with multiple studies demonstrating high local control (~85%–100%) at 2 years and low rates of Grade ≥ 3 adverse events [10]. Studies directly comparing the efficacy of SABR with TACE are limited; several small studies have shown similar or improved efficacy of SABR versus TACE, with lower rates of toxicities [10, 13, 14, 15, 16, 17, 18, 19]. SABR in combination with TACE or immunotherapy has also shown promising results, with one study showing significantly improved progression‐free survival (PFS) with SABR plus immunotherapy vs. TACE at 2 years (77.8% vs. 2.1%, *p* < 0.001) [18, 20, 21, 22]. Recently updated international guidelines now recognise the role of SABR as an alternative to ablative or transarterial therapies in very‐early‐to‐intermediate‐stage HCC, [6, 23] and the results of this survey suggest SABR use will continue to increase in Australia.

The long‐term prognosis for HCC remains poor, with a five‐year survival rate in Australia of less than 25% [1]. One of the main reasons for the poor prognosis is high recurrence rates of up to 70%, even following curative‐intent therapy [6]. As such, there is a strong clinical need for effective adjuvant therapies to reduce the risk of HCC recurrence. Most specialists agreed there are insufficient data on adjuvant systemic therapy; however, more than 30% of specialists support a potential role for adjuvant systemic therapy in patients at high risk of recurrence, particularly adjuvant immunotherapy due to its lower impact on quality of life compared with tyrosine kinase inhibitors (TKIs).

HCC guidelines do not currently recommend the routine use of adjuvant TKIs such as sorafenib or lenvatinib due to the lack of evidence of benefit [3, 4, 6]. A number of trials investigating adjuvant immunotherapy in HCC are underway, notably the IMbrave050 study, a Phase 3 trial of atezolizumab‐bevacizumab in patients with HCC at high risk of recurrence following curative resection or ablation [7]. IMbrave050 is the first study to show improved outcomes with adjuvant therapy in HCC, improving recurrence‐free survival (RFS) vs. active surveillance (HR 0.72 [adjusted 95% CI 0.53–0.98]; *p* = 0·012) at a median follow‐up of 17.4 months [7]. However, in a recently released updated analysis, the initial RFS benefit with atezolizumab‐bevacizumab vs. active surveillance was not sustained, with the OS benefit remaining immature but showing numerical improvement from the first interim analysis [24]. Nevertheless, the 2023 AASLD HCC guidelines include a strong recommendation for adjuvant immune checkpoint inhibitor (ICI) systemic therapy in patients at high risk of recurrence after hepatic resection or ablation, based on these data and expert opinion [6]. Other similar ongoing Phase 3 trials include EMERALD‐2 (durvalumab +/− bevacizumab), CheckMate‐9DX (nivolumab), and KEYNOTE‐937 (pembrolizumab) [25, 26, 27]. Data from these trials will provide key insights into the role of adjuvant immunotherapy in HCC and based on the promising results of IMbrave050 and the clear unmet need identified by specialists in this study, may drive increasing use in early‐ to intermediate‐stage HCC in Australia and adoption into Australian guidelines.

Australian and international guidelines recommend TACE as first‐line therapy for patients with BCLC‐B HCC, with systemic therapy reserved for when TACE is contraindicated or unsuitable [3, 4, 6]. However, patients with intermediate‐stage HCC represent a very heterogeneous group, and many do not benefit from TACE [11]. The 2022 updated BCLC guidelines recognize this heterogeneity and have stratified intermediate‐stage HCC into three groups of patients according to tumor burden and liver function, with ICI systemic therapy recommended first‐line for patients with diffuse, infiltrative, extensive bilobar liver involvement [5]. In this study, more than 60% of specialists who have expertise in this area consider systemic therapy first line for patients with large HCC and high tumor burden. Most specialists agreed there is a potential role for systemic therapy in intermediate‐stage HCC, but they wanted more data to define its role in this setting, particularly in terms of combination therapy with LRT.

The success of immunotherapies such as atezolizumab‐bevacizumab and durvalumab‐tremelimumab in advanced HCC has raised the question of whether the same benefits will translate to intermediate‐stage HCC [28]. There are a number of studies examining the combination of systemic therapy with LRT in intermediate‐stage HCC [28, 29]. While most studies evaluating sorafenib combined with TACE have failed to demonstrate a benefit, the Phase 3 EMERALD‐1 trial showed significantly improved median progression‐free survival (mPFS) with durvalumab + bevacizumab + TACE vs. TACE alone (15.0 vs. 8.2 months; HR 0.77; 95% CI 0.61–0.98; *p* = 0.032) [28, 30]. Other trials of immunotherapy combined with TACE are currently underway, including EMERALD‐3 (TACE with or without durvalumab + tremelimumab +/− lenvatinib) and LEAP‐012 (TACE with or without pembrolizumab + lenvatinib) [8, 31]. If results from these trials are also positive, combination immunotherapy with TACE may eventually become the standard of care.

This study has limitations inherent to its design. The reliance on a survey of a limited pool of specialists, albeit with significant expertise in managing HCC, introduces potential selection bias, as it potentially overlooks the perspectives of the broader population of Australian specialists involved in HCC management. Additionally, the analysis of only four complex real‐world HCC cases limits the generalisability of findings to a wider range of clinical presentations. Furthermore, the survey platform and facilitated discussions may have resulted in a response bias, with specialists who hold strong opinions on the topic being more likely to participate. Finally, many specialists changed their views on their preferred treatment approach during post‐survey discussions. While this reflects what occurs during an actual MDT meeting, the survey was limited in its ability to fully capture the nuances of decision‐making in HCC management.

In conclusion, this survey demonstrates that Australian treatment patterns for very early to intermediate stage HCC are largely consistent with HCC guidelines but are evolving along with the rapidly advancing treatment landscape. The increasing number of available options for managing HCC means that more complex clinical decisions are required, particularly in terms of the use of systemic therapy at earlier stages of HCC. There is growing interest in SABR as an alternative LRT and immunotherapies used as adjuvant or combination therapy in this patient group, due to emerging data showing improved patient outcomes. More data are required to define the role of SABR and systemic therapies in very early to intermediate stage HCC before widespread adoption as standard of care in Australia.

## Author Contributions

S.K.R., N.S., S.I.S., J.K.O., M.W. and A.W. contributed to the study conception and design. Material preparation, data collection and analysis were performed by Stuart Roberts, Ammar Majeed, and Kiran Rasaratnam. The first draft of the manuscript was written by Stuart Roberts with the assistance of the medical writer, Alisa Knapman, and all authors commented on previous versions of the manuscript. All authors read and approved the final manuscript.

## Conflicts of Interest

S.K.R., N.S., S.I.S., J.K.O., M.W., and A.W. received an honorarium from Astra Zeneca for their involvement on the steering committee that guided this project. A.M. received an honorarium for the statistical analysis. S.K.R. is a consultant to Astra Zeneca Australia. The content of this manuscript does not include any direct or indirect mention of any specific therapy attributable to the sponsor.

## Supporting information


Table S1.

Table S2.

## Data Availability

The dataset is available upon request to the corresponding author.
