# Supplementary material for: National Survey of Real‐World Australian Treatment Patterns for Patients With Very‐Early‐To Intermediate‐Stage Hepatocellular Carcinoma
Source: Cancer Med. 2025 Feb 28;14(5):e70722. doi: 10.1002/cam4.70722 (PMC11868786; doi:10.1002/cam4.70722)
Supplement: Supplementary file 1 — Table S1. [file CAM4-14-e70722-s001.docx]

**Supplementary Table 1**. **List of attendees by specialist group across all state-based round-table meetings conducted in Australia**.

| **Name** | **Hospital** |
| --- | --- |
| **Gastroenterologists/hepatologists** | |
| Prof Stuart Roberts | The Alfred Hospital |
| Prof John Olynyk | Fiona Stanley Hospital |
| Prof Nicholas Shackel | Launceston General Hospital |
| Dr Marnie Wood | Royal Brisbane and Women’s Hospital |
| A/prof Simone Strasser | Royal Prince Alfred Hospital |
| Prof Alan Wigg | Flinders Medical Centre |
| A/Prof Anouk Dev | Monash Health |
| A/Prof John Lubel | Alfred Health |
| A/Prof Avik Majumdar | Austin Health |
| Dr Marno Ryan | St Vincent’s Hospital |
| Prof Alex Thompson | St Vincent’s Hospital |
| Dr Steve Bollipo | John Hunter Hospital |
| Dr Judy Chiou | Gosford Hospital |
| Dr Scott Davison | Liverpool Hospital |
| Prof Jacob George | Westmead Hospital |
| Dr Ken Liu | Royal Prince Alfred Hospital |
| Dr Sarah Walker | Canberra Hospital |
| Dr Briohny Smith | Sir Charles Gairdner Hospital |
| Professor Gerry MacQuillan | Sir Charles Gairdner Hospital |
| Dr Justin Chin | Royal Perth Hospital |
| Dr Michael Wallace | Sir Charles Gairdner Hospital |
| Dr Oliver Duncan | Fiona Stanley Hospital |
| Dr Raj Uchila | Fiona Stanley Hospital |
| Dr Simon Hazeldine | Fiona Stanley Hospital |
| Dr Tim Mitchell | Royal Perth Hospital |
| Dr Wendy Cheng | Royal Perth Hospital |
| Dr Melita Andelkovic | Princess Alexandra Hospital |
| Dr Alicia Braund | Gold Coast University Hospital |
| Dr Enoka Gonsalkorala | Royal Brisbane and Women’s Hospital |
| Dr Rohit Gupta | Sunshine Coast University Hospital |
| Dr Rozemary Karamatic | The Townsville Hospital |
| Prof Barbara Leggett | Royal Brisbane and Women’s Hospital |
| Prof James O’Beirne | Sunshine Coast University Hospital |
| Dr Riaz Shaik | Redcliffe Hospital |
| Dr Andrew Buckle | Launceston General Hospital |
| Dr Chris Middleton | Royal Hobart Hospital |
| Dr Albert Nwaba | Northwest Private Hospital |
| Dr Mark Wilson | Royal Hobart Hospital |
| Dr Asif Chinnaratha | Lyell McEwin Hospital |
| Dr Dep Huynh | Queen Elizabeth Hospital |
| A/Prof Kate Muller | Royal Adelaide Hospital |
| **Interventional radiologists** | |
| Dr Vivek Ramachandran | Eastern Health |
| Dr Manfred Spanger | Eastern Health |
| Dr John Vrazas | St Vincent’s Hospital |
| Dr Jonathan Tibballs | Fiona Stanley Hospital​ |
| Dr Matthys VanWyk | Fiona Stanley Hospital |
| Dr Sam Davis | Royal Brisbane and Women’s Hospital |
| Dr Nigel Mott | Royal Brisbane and Women’s Hospital |
| Dr Andrew Halliday | Royal Hobart Hospital |
| **Radiation oncologists** | |
| Dr Sarat Chander | Peter MacCallum Cancer Centre |
| Dr Julie Chu | Peter MacCallum Cancer Centre |
| Dr Richard Khor | Austin Health |
| Dr Michael Ng | St Vincent’s Hospital |
| Dr Roya Merie | Concord Hospital |
| Dr Colin Tang | Sir Charles Gairdner Hospital |
| Dr Dominique Lee | Princess Alexandra Hospital |
| A/Prof David Pryor | Princess Alexandra Hospital |
| Dr Raghu Gowdah | Royal Adelaide Hospital |
| A/Prof Hien Le | Royal Adelaide Hospital |
| **Medical oncologists** | |
| Dr Geoff Chong | Austin Health |
| A/Prof Lara Lipton | Western Health, Cabrini |
| Dr Ankit Jain | Canberra Hospital |
| Dr Winston Liauw | Royal North Shore |
| Dr Bella Nguyen | Fiona Stanley Hospital |
| Dr Vlad Andelkovic | Princess Alexandra Hospital |
| A/Prof Amitesh Roy | Flinders Private |
| **Hepatobiliary surgeons** | |
| A/Prof Lingjun Mou | Sir Charles Gairdner Hospital |
| Dr Suresh Navadgi | Royal Perth Hospital |
| Dr Kay Bowers | Alfred Health |
| Dr Graham Starkey | Austin Health |
| Dr Nick Butler | Princess Alexandra Hospital |
| Dr Rob Bohmer | Royal Hobart Hospital |
| Dr Alistair Rowcroft | Royal Melbourne Hospital |
| Dr Mark Brooksmith | Flinders Medical Centre |
| **Hepatoma nurses** | |
| Vince Fragomeli | Nepean Hospital |
| Francisca Neveu-Coble | Westmead Hospital |
| Crystal Connelly | Fiona Stanley Hospital |
| Loveinder Madahar | Royal Perth Hospital​ |
| Marcelle Perrin | Fiona Stanley Hospital​ |
| Olivia Cullen | Royal Brisbane and Women’s Hospital |
| Kylie Bragg | Flinders Medical Centre |
| Bin Chen | Elizabeth Family Health |
| Kerry Hardy | Royal Hobart Hospital |
| Kartika Subagio | Royal Hobart Hospital |
| Tracy Young | Launceston General Hospital​ |
| **HCC pathologist** | |
| Prof Catriona McLean | Alfred Health |
