# Supplementary material for: National Survey of Real‐World Australian Treatment Patterns for Patients With Very‐Early‐To Intermediate‐Stage Hepatocellular Carcinoma
Source: Cancer Med. 2025 Feb 28;14(5):e70722. doi: 10.1002/cam4.70722 (PMC11868786; doi:10.1002/cam4.70722)
Supplement: Supplementary file 2 — Table S2. [file CAM4-14-e70722-s002.docx]

| **Treatment modality, n (%)** | **Gastroenterologist/ hepatologist** | **Other** | **Total** |
| --- | --- | --- | --- |
| Sequential cTACE to both lobes | 0 | 0 | 0 |
| Sequential DEB-TACE to both lobes | 0 | 0 | 0 |
| cTACE to both lobes | 0 | 0 | 0 |
| Sequential SIRT to both lobes | 0 | 0 | 0 |
| SIRT to both lobes | 0 | 0 | 0 |
| Systemic therapy | 7 (23%) | 8 (21%) | 15 (22%) |
| Best supportive care | **23 (77%)** | **30 (79%)** | **53 (78%)** |
| Abstain from voting | 4 | 9 | 13 |
| Total respondents, N | 30 | 38 | 68 |

**Supplementary Table 2: Management of significant bi-lobar (BCLC B) HCC in patient with more advanced (eg. Child-Pugh B8) liver disease**

cTACE = conventional transarterial chemoembolisation; DEB-TACE = drug eluting beads transarterial chemoembolisation; SIRT = selective internal radiation therapy
